# Mesenchymal stem cells ameliorate lipid metabolism through reducing mitochondrial damage of hepatocytes in the treatment of post-hepatectomy liver failure

**DOI:** 10.1038/s41419-020-03374-0

**Published:** 2021-01-21

**Authors:** Jing-lin Wang, Hao-ran Ding, Chen-yan Pan, Xiao-lei Shi, Hao-zhen Ren

**Affiliations:** 1 Department of Hepatobiliary Surgery, Affiliated Drum Tower Hospital of Nanjing University Medical School, Nanjing, Jiangsu Province China; 2 Department of Hepatobiliary Surgery, Nanjing Drum Tower Hospital Clinical College of Nanjing Medical University, Nanjing, Jiangsu Province China; 3 Department of Hepatobiliary Surgery, Nanjing University of Chinese Medicine, Nanjing, Jiangsu Province China

**Keywords:** Mesenchymal stem cells, Hepatotoxicity

## Abstract

Hepatectomy is an effective therapeutic strategy for many benign and malignant liver diseases, while the complexity of liver anatomy and the difficulty of operation lead to complications after hepatectomy. Among them, post-hepatectomy liver failure (PHLF) is the main factor threatening the life of patients. At present, liver transplantation is an effective approach for PHLF. However, the application of liver transplantation has been largely limited due to the shortage of donors and the high cost of such operation. Therefore, it is urgently necessary to develop a new treatment for PHLF. Mesenchymal stem cells (MSCs) have become a new treatment regimen for liver diseases because of their easy access and low immunogenicity. Our study found that there were some subtle connections between MSCs and liver lipid metabolism in the PHLF model. We used MSC transplantation to treat PHLF induced by 90% hepatectomy. MSC transplantation could restore the mitochondrial function, promote the β-oxidation of fatty acid (FA), and reduce the lipid accumulation of hepatocytes. In addition, interleukin 10 (IL-10), a cytokine with immunoregulatory function, had an important role in lipid metabolism. We also found that MSCs transplantation activated the mammalian target of rapamycin (mTOR) pathway. Therefore, we explored the relationship between mitochondrial damage and lipid metabolism abnormality or PHLF. MSCs improved mitochondrial function and corrected abnormal lipid metabolism by affecting the mTOR pathway in the treatment of PHLF. Collectively, MSC transplantation could be used as a potential treatment for PHLF.

## Introduction

The liver disease remains a critical threat to human health. Surgical resection remains an effective treatment for some benign and malignant liver diseases. Because of the complexity of liver anatomy and the difficulty of liver surgery, the complications after major hepatectomy become inevitable. Despite the continuous progress of medical support and postoperative care, liver failure after hepatectomy is still the main cause of death in patients undergoing hepatectomy^1^. The inevitable metabolic abnormality after hepatectomy is still an important cause of post-hepatectomy liver failure (PHLF), which is an important factor threatening the life of patients. Although the liver has a strong ability of regeneration, in order to ensure the regeneration of the remaining liver, at least 30% of the liver needs to be preserved after surgical resection^2^. PHLF refers to that the remaining liver after hepatectomy is not enough to meet the clinical symptoms required by the body, which is characterized by liver necrosis, rapid elevation of serum transaminase, coagulation dysfunction, and so on, seriously threatening the life of patients. Once serious PHLF occurs, there is no other effective treatment except for liver transplantation.

Mesenchymal stem cells (MSCs) are widely studied in the fields of immunology and regenerative medicine because of their wide sources, easy access, immune regulatory role, and promotive effect on tissue regeneration^3,4^. Previous studies have shown that MSCs have an important role in immunoregulation and tissue repair by interacting with inflammatory cells^5^. MSCs transplantation is a promising therapeutic approach for liver diseases.

As an important metabolic organ, the liver is involved in lipid metabolism, glucose metabolism, and other metabolisms^6^. Maintaining a balanced lipid metabolism in hepatocytes can significantly reduce liver damage and promote the recovery of liver function^7^. Previous studies have confirmed that within 6 h after liver surgery, liver cells have abnormal metabolism and abnormal lipid accumulation, which eventually leads to liver injury and liver failure^8^. Therefore, a therapeutic strategy, which can regulate the lipid homeostasis of hepatocytes after hepatectomy, will effectively reduce the risk of PHLF. Hepatocytes are the main place of lipid metabolism in the human body. Normal lipid metabolism in hepatocytes mainly includes four parts as follows: synthesis, uptake, efflux and oxidation. These processes are precisely regulated by the body to maintain the homeostasis of intracellular lipid levels. Extracellular triglyceride (TG) and fatty acid (FA) enter the hepatocytes under the action of FA transferase (CD36), and part of them generate ATP through mitochondrial β-oxidation mediated by carnitine palmitoyltransferase 1a (CPT1A) and hydroxyacyl-CoA dehydrogenase/3-ketoacyl-CoA thiolase/enoyl-CoA hydratase alpha/beta subunit (HadhA/B), while TG and FA without oxidative respiration are stored in hepatocytes in the form of lipid droplets under the action of stearoyl-CoA desaturase 1a (Scd1a), acetyl-CoA carboxylase (ACC) and FA synthase (FASN)^9–11^. Considering the liver as a metabolic organ, how to correct the metabolic disorder after liver surgery is the core of successful treatment or even avoidance of PHLF.

Liu has found that MSCs can also regulate lipid metabolism in adipose tissue^12^. MSCs can induce the activation of uncoupling protein-1 (UCP-1) and PRD1-BF1-RIZ1 homologous domains to promote lipid catabolism^12^. However, only very few studies have investigated the effect of MSCs on liver lipid metabolism after hepatectomy. It has shown exciting therapeutic effects in drug-induced liver failure^13^, while only a few studies have been reported in liver surgical models. Major hepatectomy can induce severe steatosis and lipotoxicity^14,15^. In the present study, we aimed to verify the therapeutic effect of MSCs on PHLF and explore the role of MSCs in lipid metabolism after hepatectomy.

## Methods

### Animals

Sprague–Dawley (SD) rats were purchased from the Animal Experimental Base of Nanjing Drum Tower Hospital Affiliated to Nanjing University. Rats of 8–12 weeks old (weighing 200–300 g) were selected as the study subjects to receive hepatectomy and/or MSC transplantation. The animal-related experiments were approved by the Institutional Animal Care and Use Committee of Nanjing University, Nanjing, China (No. 20180701).

### Isolation and culture of MSCs

MSCs were obtained from SD rats (about 4 weeks, 120–140 g) according to the previously described procedures^16^. Briefly, the femur and tibia were carefully dissected from SD rats, followed by separation of the soft tissue around the bone. Then phosphate-buffered saline (PBS) was used to wash the marrow cavity to obtain cells in the marrow cavity. Cell precipitation was obtained by centrifugation at 1200 rpm for 5 min, and the obtained cells were maintained in SD-MSC conditional medium (Cyagen Bioscience Inc., China) supplemented with 10% fetal bovine serum (Sciencell, San Diego, CA, United States), 100 mg/mL streptomycin and 100 U/mL penicillin at 37 °C containing 5% CO_2_. After 24 h of incubation, unattached cells were discarded, and the adherent cells were cultured and passaged at an appropriate time.

In order to inhibit or improve the ability of MSCs to secrete interleukin 10 (IL-10), the short hairpin RNA (shRNA) against IL-10 or a lentivirus harboring IL-10 gene (GeneChem, Shanghai, China) was transfected to MSCs following the manufacturer’s instructions. The IL-10 level in the supernatant was determined by ELISA (R&D Systems, Minneapolis, MN, USA).

### Surgery and treatment

Rats were subjected to partial hepatectomy (PH) to construct the PHLF model. Briefly, 90% of hepatectomy was performed according to the previously reported procedure^17^. The rats were anesthetized by isoflurane inhalation, and then a median abdominal incision was made on the abdominal cavity. Subsequently, the ligaments around the liver were separated and disconnected. The left lobe, middle lobe, right upper lobe, and right lower lobe of the liver were ligated and successively excised with silk thread. The abdominal cavity was closed after confirming that there was no active bleeding in the abdominal cavity. In cell transplantation, the single-cell suspension (5 × 10^6^ in 1 mL PBS) was injected from the portal vein. The rats were randomly divided into five groups as follows: (1) PH + PBS group, rats underwent 90% hepatectomy and PBS injection from portal vein; (2) PH + MSCs group, rats underwent 90% hepatectomy and MSCs transplantation from portal vein; (3) PH + MSCs-IL-10(−) group, IL-10-depleted MSCs were used for cell transplantation in rats receiving 90% hepatectomy; (4) PH + MSCs-IL-10(+) group, MSCs over-expressing IL-10 were used for cell transplantation in rats receiving 90% hepatectomy; (5) PH + MSCs + RAP group, rats receiving 90% hepatectomy were given rapamycin (RAP, 1 mg/kg, every 24 h) and MSC transplantation.

### Western blotting analysis

Ripa (KeyGen Biotech, Nanjing, China) with PMSF was used to extract proteins from liver tissues or cells at 4 °C. Protein concentration was determined by BCA protein assay (KeyGen Biotech, Nanjing, China). The western blotting analysis was carried out as previously described^18^. Detailed information about antibodies used was listed in Table 1.

**Table. 1:** Antibodies for immunoblots and immunohistochemistry.

Antigene	Company
ACC	Cell Signaling Technology, 3676
FASN	Cell Signaling Technology, 3180
PPARα	Arigobio, ARG56482
CPT1a	EMD Millipore, ABS65
CROT	Abcam, ab175450
SREBP1	ImmunoWay, YT5508
SREBP2	ImmunoWay, YN0037
Drp1	Cell Signaling Technology, 8570
p-Drp1(Ser616)	Cell Signaling Technology, 3455
Fis1	Proteintech, 10956-1-AP
MFN1	Proteintech, 13798-1-AP
MFN2	Proteintech, 12186-1-AP
UCP-1	EMD Millipore, AB1426
p-MTOR (Ser2448)	Cell Signaling Technology, 5536
MTOR	Cell Signaling Technology, 2983
p-p70 S6K (T389)	Cell Signaling Technology, 9430
p70 S6K	Cell Signaling Technology, 2708
p-4E-BP1 (T37/46)	Cell Signaling Technology, 2855
4E-BP1	Cell Signaling Technology, 9644
p-AKT (Ser473)	Cell Signaling Technology, 4060
AKT	Cell Signaling Technology, 4691
RICTOR	Cell Signaling Technology, 2114
RAPTOR	Cell Signaling Technology, 2280
GAPDH	Cell Signaling Technology, 5174

### Real-time quantitative PCR (RT-qPCR)

Total RNA was extracted from rat liver using Trizol reagent (ThermoFisher Scientific). Purified RNA was reversely transcribed into cDNA using the Prime Script Reverse Transcription Reagent Kit (Takara Bio, Shiga, Japan), and RT-qPCR was performed on an ABI7500 detection system using SYBR Premix Ex Taq (Takara Bio). Sequences of primers used in the present study were given in Table 2.

**Table. 2:** The sequences of the primers.

Genes	Forward primer (5′–3′)	Reverse primer (5′–3′)
*Cpt1a*	CGCCATACTGCTGTATCGTC	ACAATGTGCCTGCTGTCCT
*Hadha*	GGGATGTGGCAGTTATTCG	CCTGATTTGGTCGTTGGC
*Hadhb*	TGGTGGAAGGTGTCCGAA	CAACAAACCCGAAAGTGCG
*Scd1*	TCCAGAGGAGGTACTACAAGCC	AAGTTTCGCCCCAGCAGT
*Acaca*	ACCAGCACTCCCGATTCA	TCCTCTAGGTCCAGCTTTACC
*Fasn*	AGCCGCCGACCAGTATAAA	GCACAGACACCTTCCCATCA
*CD36*	CTTCCAGCCAACGCCTTT	TGCACTTGCCAATGTCCAG
*GAPDH*	ACAGCAACAGGGTGGTGGAC	TTTGAGGGTGCAGCGAACTT

### Histological analysis

Liver tissue was fixed in 4% paraformaldehyde. The paraffin-embedded samples were then sectioned into slices (5-mm-thick, three from each liver). The sections were stained with hematoxylin and eosin (H&E) for pathological examination. Finally, the sections were observed under a microscope.

### Liver function and blood coagulation indexes were detected

Blood samples were collected from the abdominal aorta of rats at 6, 12, 24, 48, and 72 h post-operation. The levels of ALT, AST, and blood ammonia as well as the INR were determined with an automated biochemical analyzer (Olympus) in the Affiliated Drum Tower Hospital of Nanjing University Medical School.

### JC-10 staining and Janus green B staining

Rats liver cells were extracted following a previously established procedure. Briefly, the rats were anesthetized and then well fixed, followed by perfusion with 0.02% type IV collagenase from the portal vein for 6 min. The liver capsule was removed, and the liver was dissected. Subsequently, the liver cells were collected and filtered through a 200-mesh filter, followed by staining with JC-10 (KeyGen Biotech, Nanjing, China) working solution (37 °C, 5% CO_2_, 15 min). The stained cells were observed under a fluorescence microscope.

### Transmission electron microscopy (TEM)

The liver tissue was fixed with glutaraldehyde (2.5%), dehydrated, embedded, and solidified by conventional methods. The embedded tissue was cut into 40-nm sections and subjected to TEM (JEM-1011, Japan).

### Statistical analysis

All data were expressed by the mean ± standard deviation. All statistical analyses were conducted using the Prism software (version 6.0; GraphPad Software Inc., La Jolla, CA, United States). Two independent samples were compared using a Student’s *t*-test. *P* < 0.05 was considered statistically significant.

## Results

### MSCs transplantation reduces PHLF

**Fig. 1:**
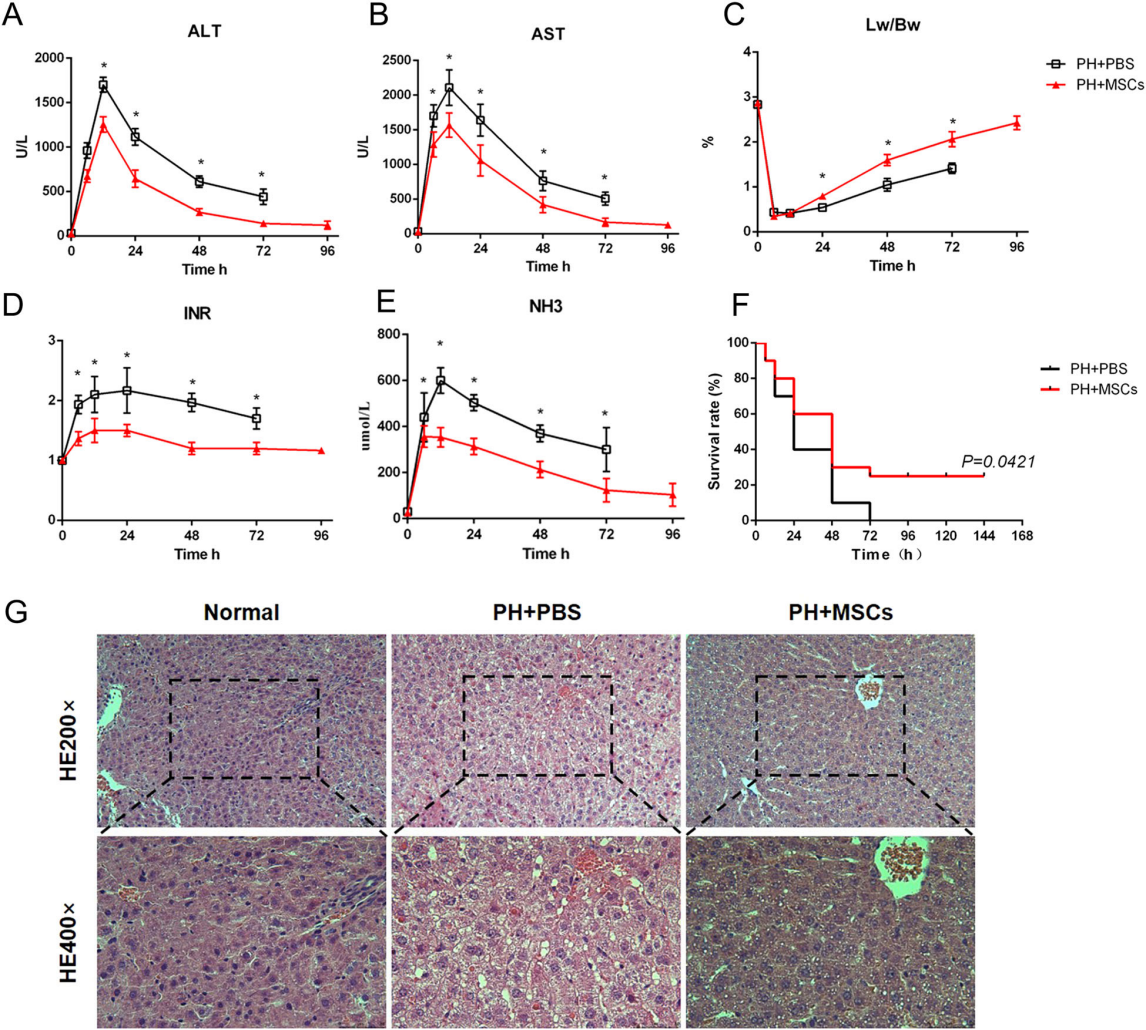
MSCs attenuate PHLF. **A**, **B**, **D**, **E** The levels of serum ALT, AST, INR, and ammonia were determined at 6, 12, 24, 48, 72, and 96 h. MSCs transplantation significantly decreased the levels of liver enzymes, INR, and ammonia after infusion. **C** Survival analysis of liver failure mice with PBS and MSCs transplantation. MSCs transplantation resulted in a significant decrease in mortality. **F** The ratio of liver to body weight. MSCs transplantation promoted liver regeneration. **G** Liver H&E staining. *P < 0.05. *t*-test, data are shown as mean standard deviation.

A PHLF model was established by 90% hepatectomy in rats. This is a classic animal model to simulate PHLF based on the previous studies^19^. The levels of liver enzymes (ALT and AST) in hepatectomized rats were sharply increased. We found that this model had a very high mortality rate. Almost no rats could survive after 90% hepatectomy without treatment. Encouragingly, the levels of ALT and AST were decreased after MSCs transplantation, and the survival rate after MSCs transplantation through portal vein was more than 20% (Fig. 1A–C). The acute damage of liver function is often accompanied by the deterioration of coagulation function and the increased level of blood ammonia. We observed that the blood clotting function of the hepatectomized rats deteriorated to a large extent, and the level of blood ammonia was also significantly increased, while such changes were improved after MSCs transplantation (Fig. 1D, E). In order to explore the liver regeneration after hepatectomy, we compared the changes in the liver weight ratio between the two groups. We found that the MSCs transplantation promoted liver regeneration, while such change was not obvious in the early stage (the first 12 h) after MSCs transplantation (Fig. 1F). Next, we assessed the residual liver injury in rats using H&E staining and found that there was swelling of the remaining liver cells and damage to the lobular structure after the operation. These injuries were improved to some extent after MSCs transplantation (Fig. 1G). Interestingly, we observed the vacuolar degeneration of hepatocytes after hepatectomy as well as small vacuoles in hepatocytes after MSCs transplantation (Fig. 1G). This finding suggested that hepatectomy was associated with hepatic steatosis. According to the serological and histological indicators, we hypothesized that MSCs transplantation might be a potential approach for the treatment of PHLF.

### Liver lipid accumulation associated with hepatectomy is reduced after MSCs transplantation

**Fig. 2:**
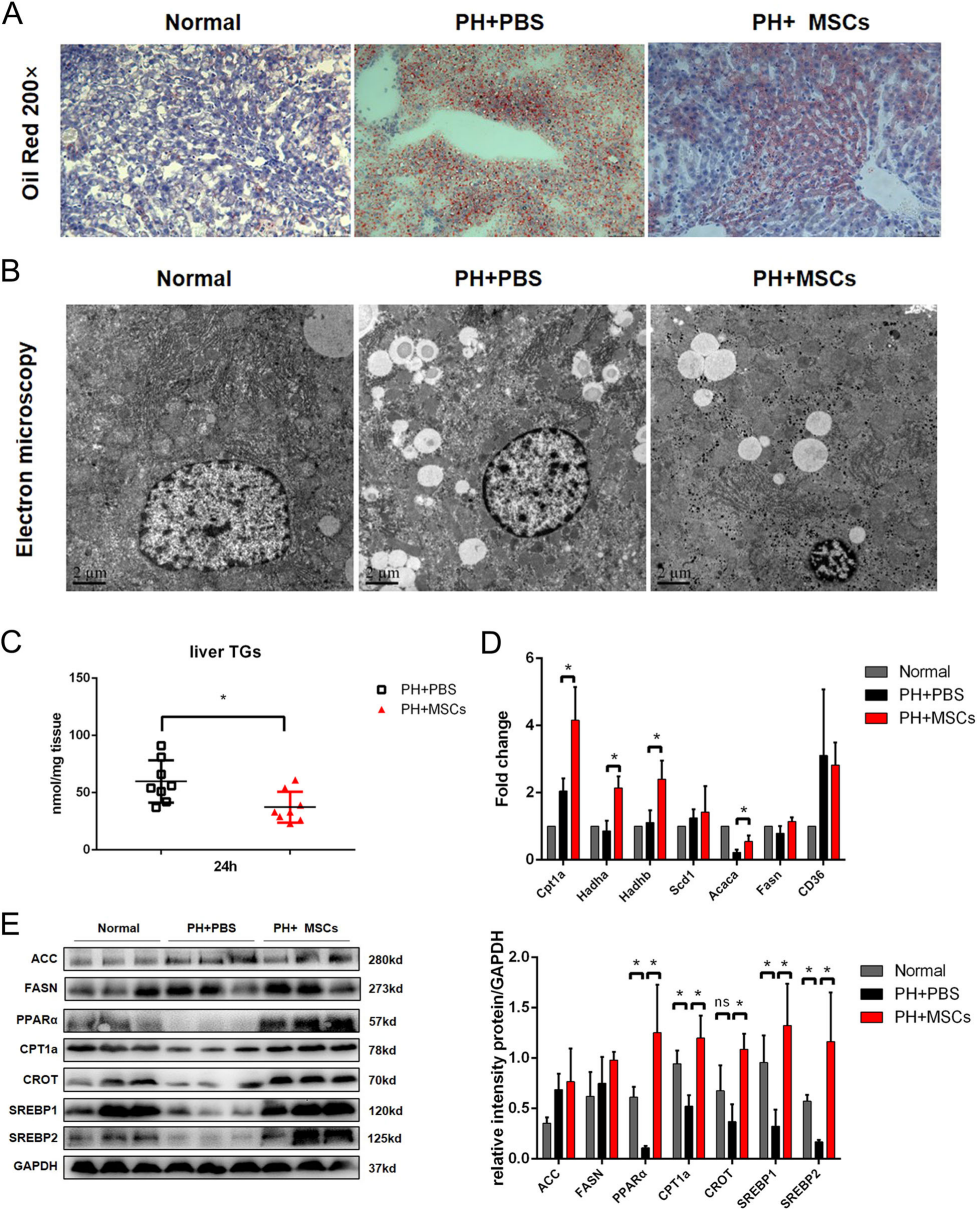
MSCs transplantation reduces lipid accumulation after hepatectomy. **A** Oil red staining of liver tissue. **B** Electron microscopy of liver tissue. **C** Detection of TG content in liver tissue. **D**, **E** Western blotting analysis and RT-PCR of liver lipid metabolism-related genes and proteins. *P < 0.05. *t*-test, data are shown as mean standard deviation.

We evaluated the lipid accumulation in the remaining hepatocytes after hepatectomy. Our data showed found that 90% hepatectomy could induce severe steatosis by oil red O staining, and severe lipid accumulation was reversed after MSCs transplantation (Fig. 2A). We further evaluated the hepatic steatosis by TEM. The results showed that 90% hepatectomy resulted in more lipid droplets to hepatocytes, and MSCs transplantation reduced the number of lipid droplets in hepatocytes, while such level was not restored to the normal situation (Fig. 2B). As expected, there was a significant reduction in TG content in the liver after MSCs treatment (Fig. 2C). In order to clarify the mechanism underlying the decrease of lipid accumulation, we detected the expressions of genes related to lipid metabolisms, such as lipid decomposition (CPT1a, Hadha, Hadhb), lipid synthesis (Scd1, Acaca, FASN), and lipid transport (CD36). The results indicated that MSCs mainly affected the process of lipid decomposition and metabolization rather than lipid synthesis and transport (Fig. 2D). In order to verify this finding, we assessed the contents of lipid metabolism-related proteins in three groups of rats. Consistent with our findings, the expressions of lipolysis-related proteins, such as peroxisome proliferator-activated receptor alpha (PPARα), CPT1a, and carnitine octanoyltransferase (CROT), were increased after MSCs transplantation, although there was no significant change in lipid synthesis-related proteins (ACC and FASN). Interestingly, the expressions of sterol-regulatory element-binding proteins (SREBP1 and SREBP2) were also restored, although the expression of downstream FASN was not significantly changed. This finding might be attributed to that MSCs reduced lipid accumulation and restored lipid metabolism (Fig. 2E). Moreover, PPARα, a key transcription factor for β-oxidation of FA, was upregulated after MSCs transplantation. In addition, the expressions of two targets (CPT1a and CROT) regulated by PPAR were also increased, indicating that MSCs transplantation could improve the function of FA β-oxidation.

### MSCs transplantation ameliorates mitochondrial damage of hepatocytes

**Fig. 3:**
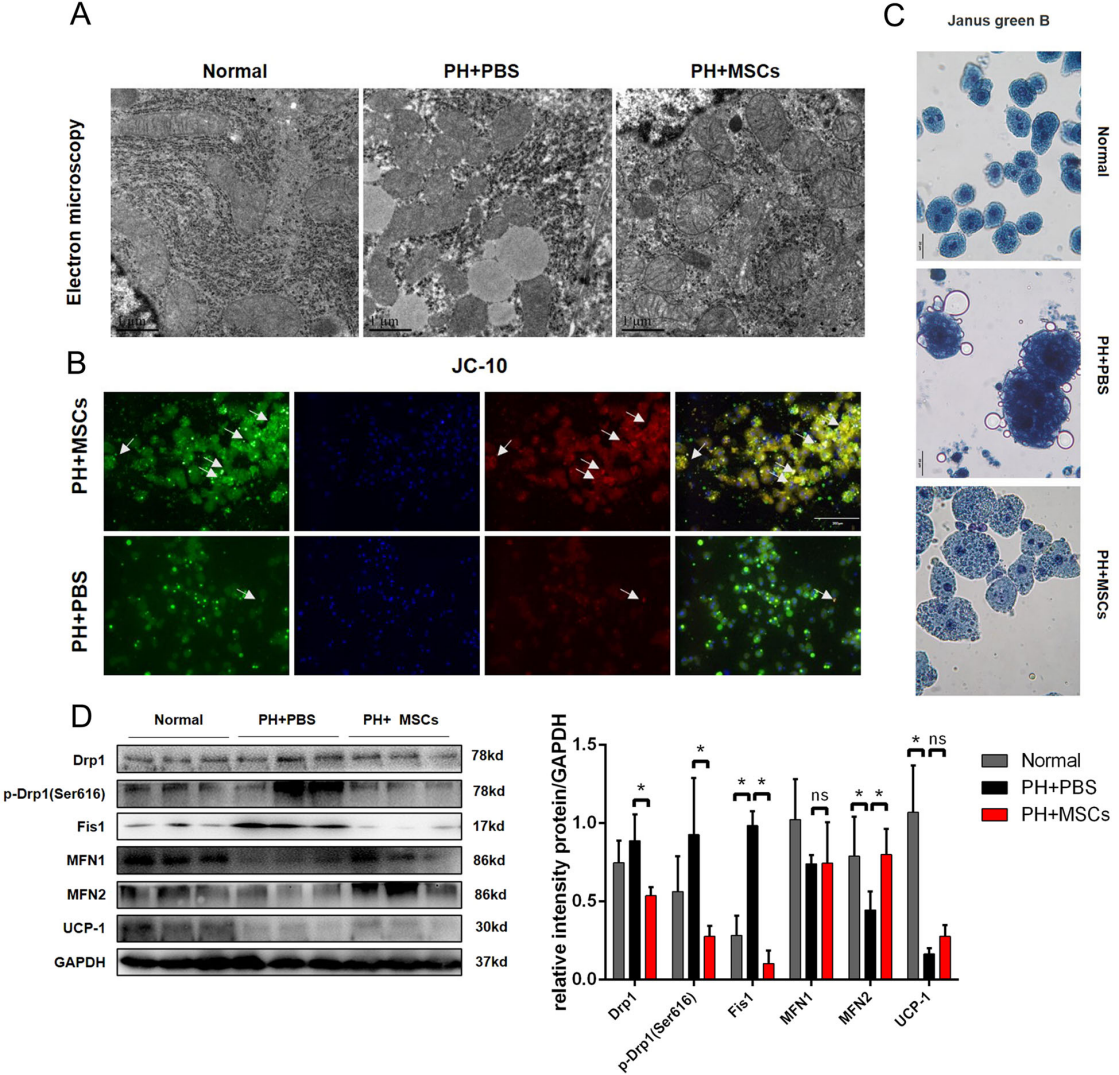
MSCs transplantation reduces mitochondrial damage in PHLF. **A** Observation of mitochondria in hepatocytes by transmission electron microscopy. **B** Observation of mitochondrial membrane potential in hepatocytes by JC-10 staining. **C** Janus green B staining. **D** Mitochondrial dynamic protein detection. *P < 0.05. *t*-test, data are shown as mean standard deviation.

Mitochondria are recognized as the main organelles, in which β oxidation of FA takes place. We examined the situation of mitochondrial damage in liver tissue using TEM. Our data showed that after 90% hepatectomy, more lipid droplets were found in the cytoplasm, the staining of the mitochondrial matrix was deepened, the matrix was disordered, the crista disappeared, and the volume became smaller. After MSCs transplantation, although the mitochondria were swollen, the morphology of mitochondria was significantly improved (Fig. 3A). In order to further evaluate the mitochondrial damage, JC-10 staining was used to detect mitochondrial membrane potential. We extracted the primary hepatocytes from the remaining liver after hepatectomy and found that the mitochondrial membrane potential was corrected after MSCs transplantation (Fig. 3B). After hepatectomy, the remaining hepatocytes were stained more deeply by Janus green B staining, which was relieved by MSCs transplantation (Fig. 3C). In order to clarify the changes of mitochondrial function in three groups of rats, we examined the expressions of mitochondrial dynamic proteins, such as dynamin-related protein-1 (Drp1), p-Drp1, fission 1 (Fis1), mitofusin 1 (MFN1) and MFN2, and mitochondrial oxidative respiratory protein (UCP-1). Our data showed that the remaining mitotic proteins (p-Drp1 and Fis1) in the liver were activated, while these proteins were downregulated after MSCs transplantation. Mitochondrion fusion-related protein (MFN2) was also improved after MSCs transplantation. These results suggested that the disruption of mitochondrial dynamic balance induced by 90% hepatectomy could be improved by MSCs transplantation (Fig. 3D). Taken together, MSCs could reduce the mitochondrial damage caused by hepatectomy.

### MSCs transplantation promotes the activation of mammalian target of rapamycin (mTOR) pathway in the remaining liver after hepatectomy

**Fig. 4:**
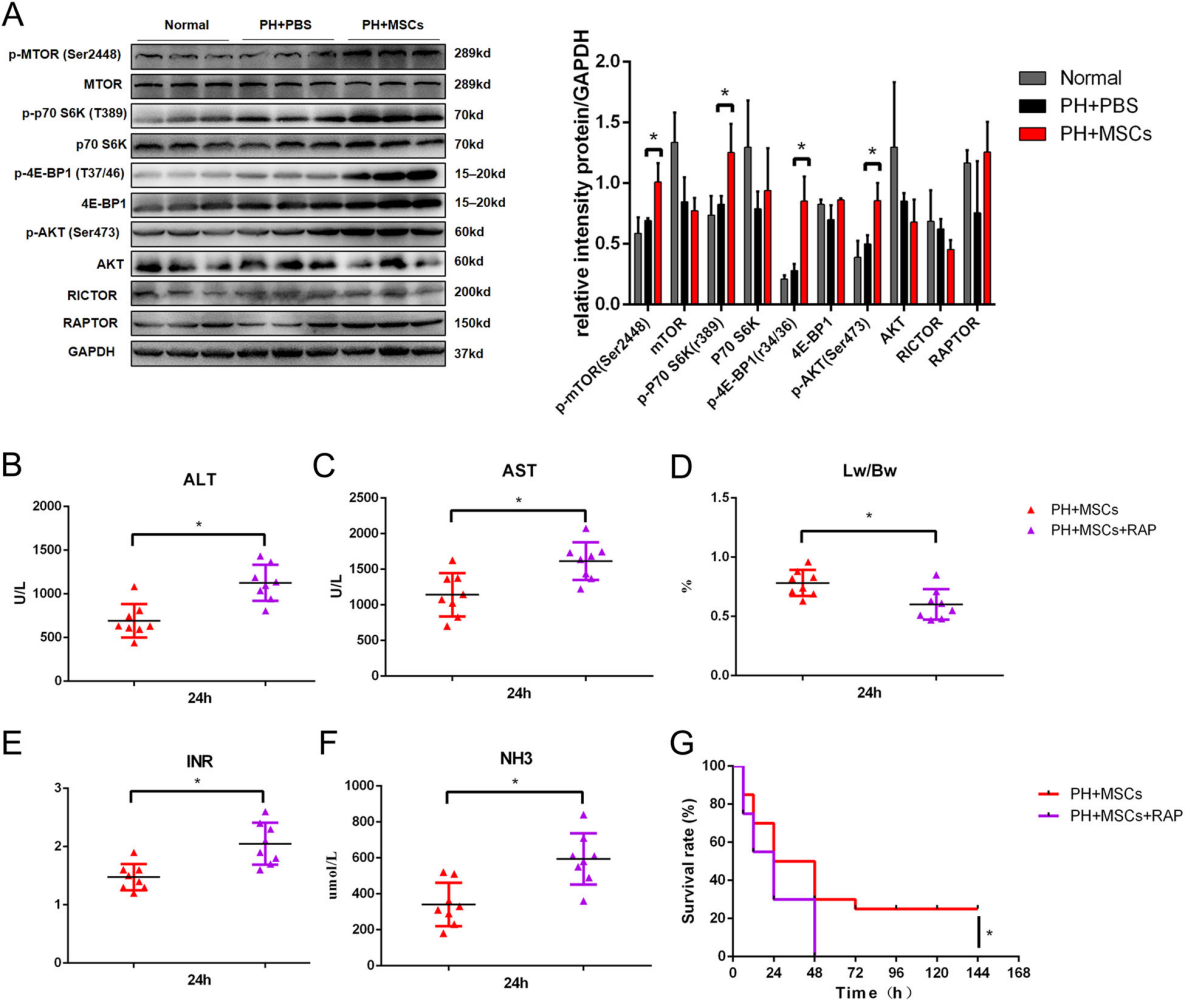
MSCs transplantation improves the mTOR pathway after hepatectomy. **A** Western blotting analysis of mTOR pathway. **B**, **C**, **E**, **F** The levels of serum ALT, AST, PT, and ammonia were determined at 24 h. **D** The ratio of liver to body weight. **G** Survival analysis of PHLF rats. *P < 0.05. *t*-test, data are shown as mean standard deviation.

**Fig. 5:**
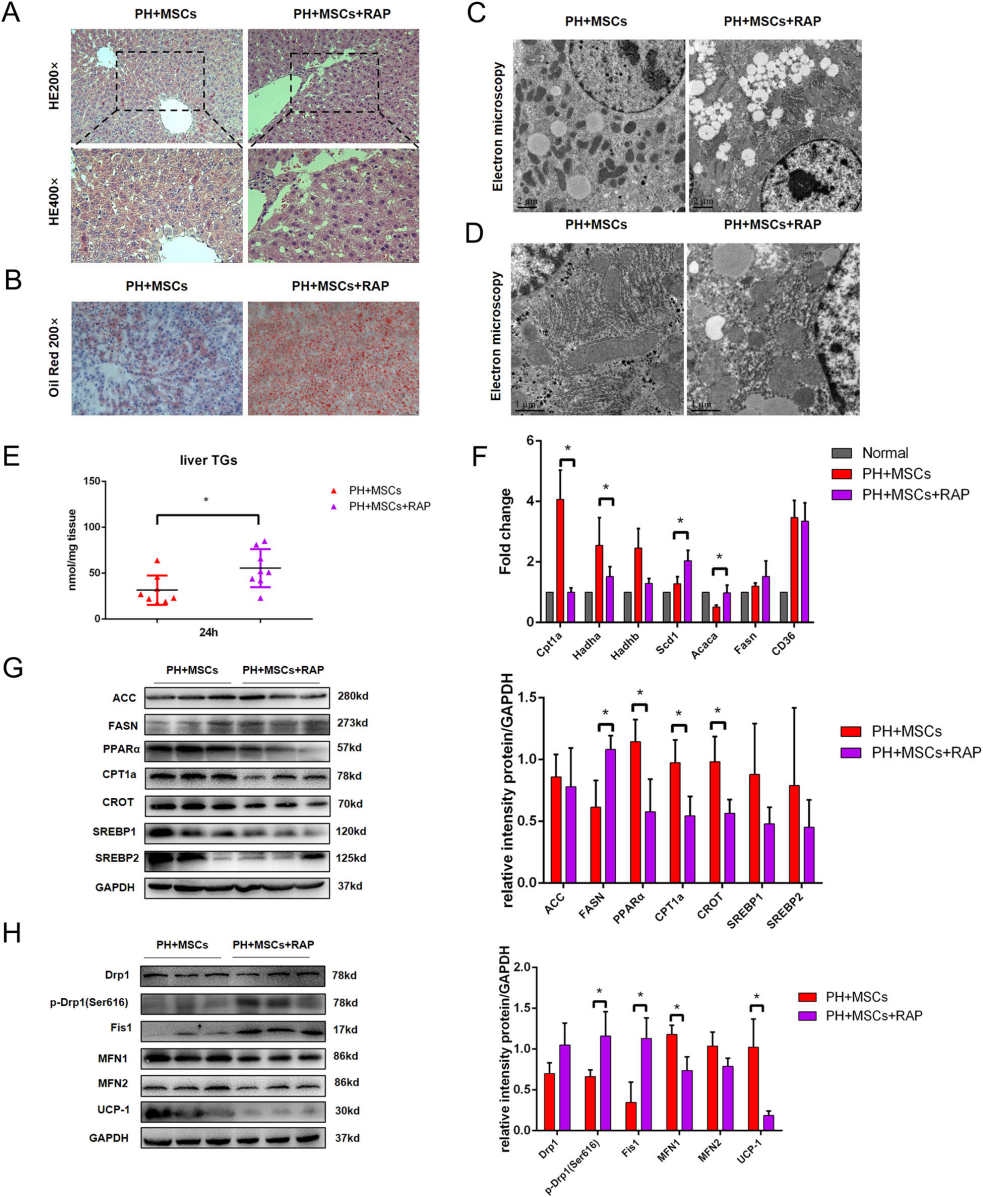
mTOR pathway is involved in the regulation of MSCs on lipid metabolism. **A** Liver H&E staining. **B** Oil red staining of liver tissue. **C**, **D** Electron microscopy of liver tissue. **E** Detection of TG content in liver tissue. **F**, **G** Western blotting analysis and RT-PCR of liver lipid metabolism-related genes and proteins. **H** Mitochondrial dynamic protein detection. *P < 0.05. *t*-test, data are shown as mean standard deviation.

As an atypical serine/threonine kinase, the mTOR pathway has an important role in regulating tissue regeneration and metabolism. We examined the expressions of mTOR-related proteins in the remaining liver of rats undergoing hepatectomy and MSCs transplantation. After 90% hepatectomy, the activation of the mTOR pathway was not significant, and there was no statistical difference. Encouragingly, MSCs transplantation could activate the mTOR pathway in the remaining liver (Fig. 4A). To verify the importance of the mTOR pathway in MSCs transplantation for PHLF, we adopted RAP to inhibit such pathway (1 mg/kg). As expected, the inhibition of the mTOR pathway hindered the therapeutic effect of MSCs. RAP administration increased the liver enzyme index after MSCs transplantation and worsened the blood coagulation and blood ammonia. The liver regeneration was inhibited after 90% hepatectomy, and the mortality of rats was increased (Fig. 4B–G). In addition, mTOR activator agonist MHY1485 (10 mg/kg) can have a certain therapeutic effect on PHLF. In terms of survival rate, the effect of MHY1485 is not as effective as using MSCs directly, which may be related to other functions of MSCs (Supplementary Fig. 1A). We found that MSCs transplantation activated the mTOR pathway, and the activation of such pathway had a crucial role in the recovery of PHLF. As a pathway closely related to liver lipid metabolism, mTOR might be involved in the effect of MSCs on mitochondria and lipid metabolism. H&E staining showed that inhibition of the mTOR pathway aggravated the vacuolar degeneration of hepatocytes after hepatectomy (Fig. 5A). Moreover, TEM and oil red O staining confirmed that RAP caused more lipid accumulation, which reversed the effect of MSCs on the reduction of lipotoxicity induced by hepatectomy (Fig. 5B, C). We then examined the mitochondria by TEM. The results showed that RAP could inhibit mitochondrial function (Fig. 5D). The inhibition of the mTOR pathway more significantly exacerbated the lipid accumulation, lipid metabolism disorder, and mitochondrial dynamic balance disruption (Fig. 5E–H). These data indicated that the mTOR pathway had an important role in the regulation of lipid metabolism after hepatectomy, further revealing that the mTOR pathway was an important hub between regeneration and metabolism.

### MSCs ameliorates PHLF by secreting IL-10

**Fig. 6:**
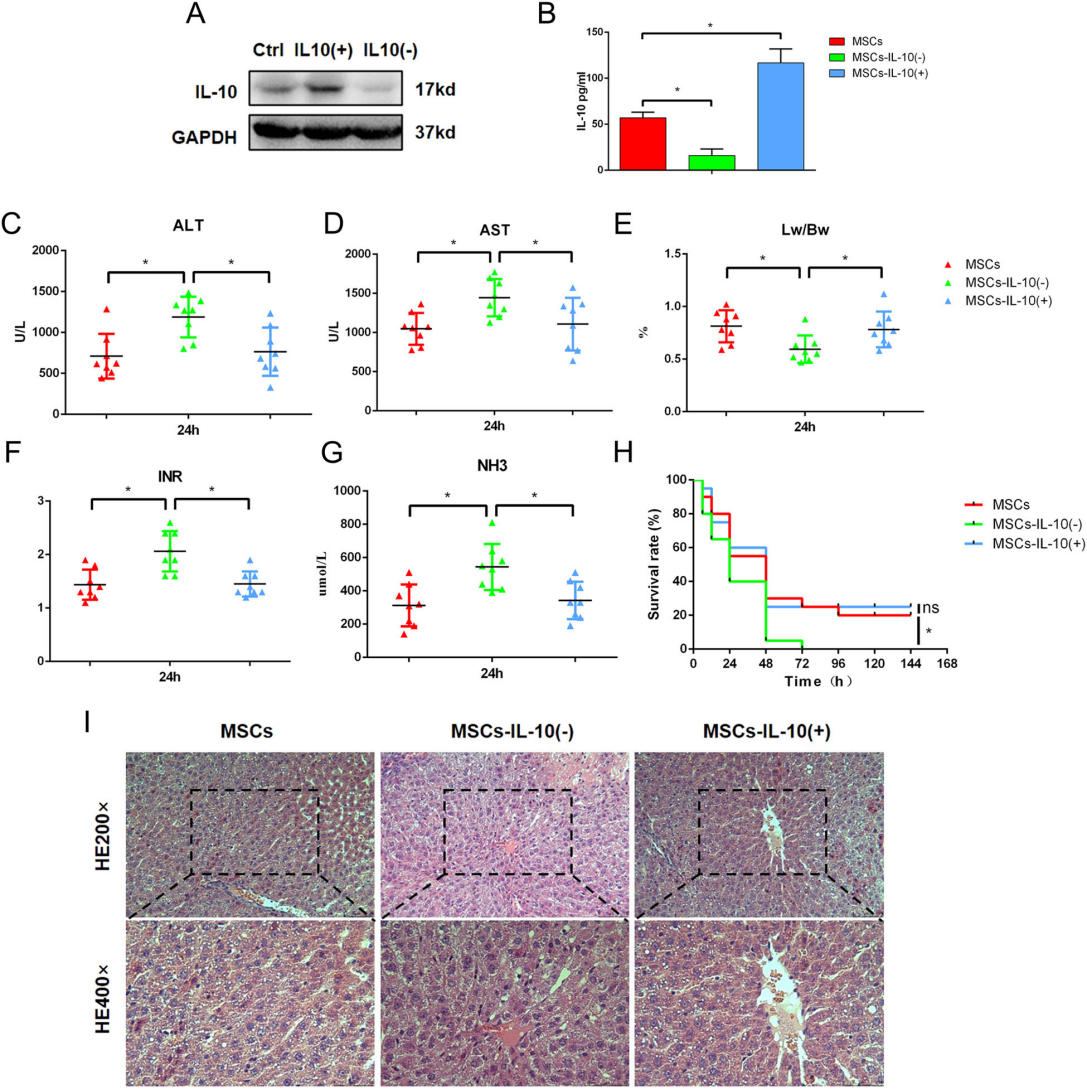
The important role of IL-10 in MSCs transplantation. **A**, **B** Construction and verification of MSCs-IL-10(−) and MSCs-IL-10(+). **C**, **D**, **F**, **G** The levels of serum ALT, AST, INR, and ammonia were determined at 24 h. **E** Survival analysis of PHLF rats. **H** The ratio of liver to body weight. **I** Liver H&E staining. *P < 0.05. *t*-test, data are shown as mean standard deviation.

The therapeutic effect of MSCs depends on paracrine cytokines, including IL-10, PGE2, and IL-6^20,21^. IL-10 is a cytokine that can regulate the growth and differentiation of innate immune cells. At present, studies have confirmed that IL-10 can regulate cell proliferation, apoptosis, angiogenesis, and inflammation^22,23^. Some studies have shown that the expression of IL-10 in serum is related to the occurrence of lipid metabolism disorder^24^. IL-10 is the key link between inflammatory factors and lipid metabolism. By detecting the cytokine content in the liver of 24 h, we also observed that IL-10 was significantly different. It seems that MSC does not have a therapeutic role through lipophagy (Supplementary Fig. 1B). To explore the role of IL-10 in MSCs transplantation, MSCs lines over-expressing or with depleted IL-10 were established by using lentivirus (Fig. 6A, B). Then the rats were randomly divided into three groups and treated with MSCs, MSCs with depleted IL-10, or MSCs over-expressing IL-10 after 90% hepatectomy. Through the detection of serum markers (ALT, AST, INR, and NH3), we found that IL-10 had a key role in MSCs transplantation therapy. The depletion of IL-10 reversed the therapeutic effect of MSCs on PHLF, showing reduced survival rate and decreased ratio of liver to body (Fig. 6C–H). Surprisingly, MSCs over-expressing IL-10 did not show a better therapeutic effect. The expression of IL-10 was closely related to the pathological damage of the remaining liver. When MSCs with depleted IL-10 were used in the treatment of PHLF, the hepatocytes showed swelling and vacuolar degeneration, which reversed the therapeutic effect of MSC transplantation (Fig. 6I). Therefore, IL-10 was a key cytokine for MSCs, which had a therapeutic role and might be related to lipid metabolism.

### MSCs improve liver lipid metabolism after hepatectomy through IL-10 signaling

**Fig. 7:**
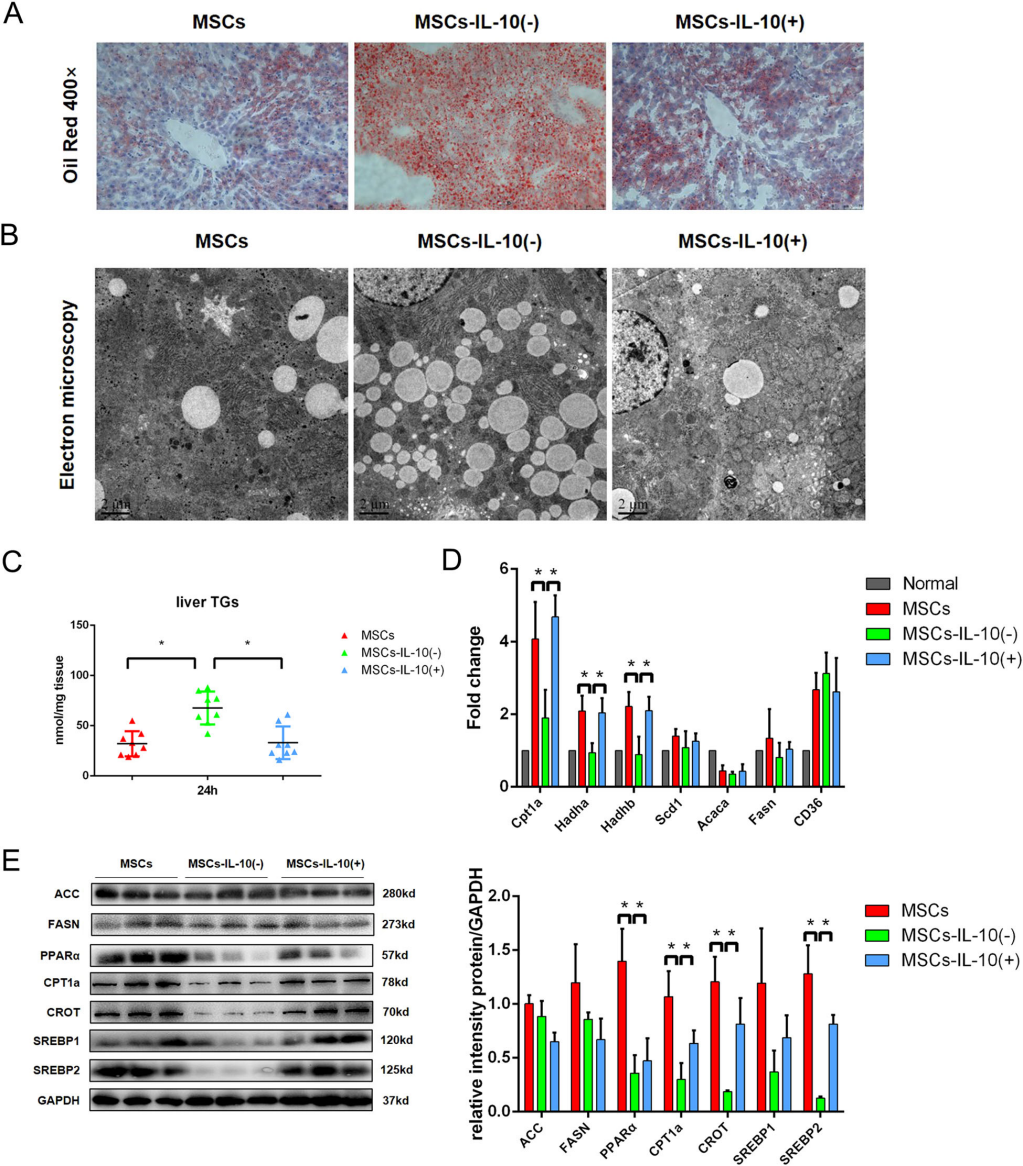
MSCs affect lipid metabolism in PHLF by secreting IL-10. **A** Oil red staining of liver tissue. **B** Electron microscopy of liver tissue. **C** Detection of TG content in liver tissue. **D**, **E** Western blotting analysis and RT-PCR of liver lipid metabolism-related genes and proteins. *P < 0.05. *t*-test, data are shown as mean standard deviation.

**Fig. 8:**
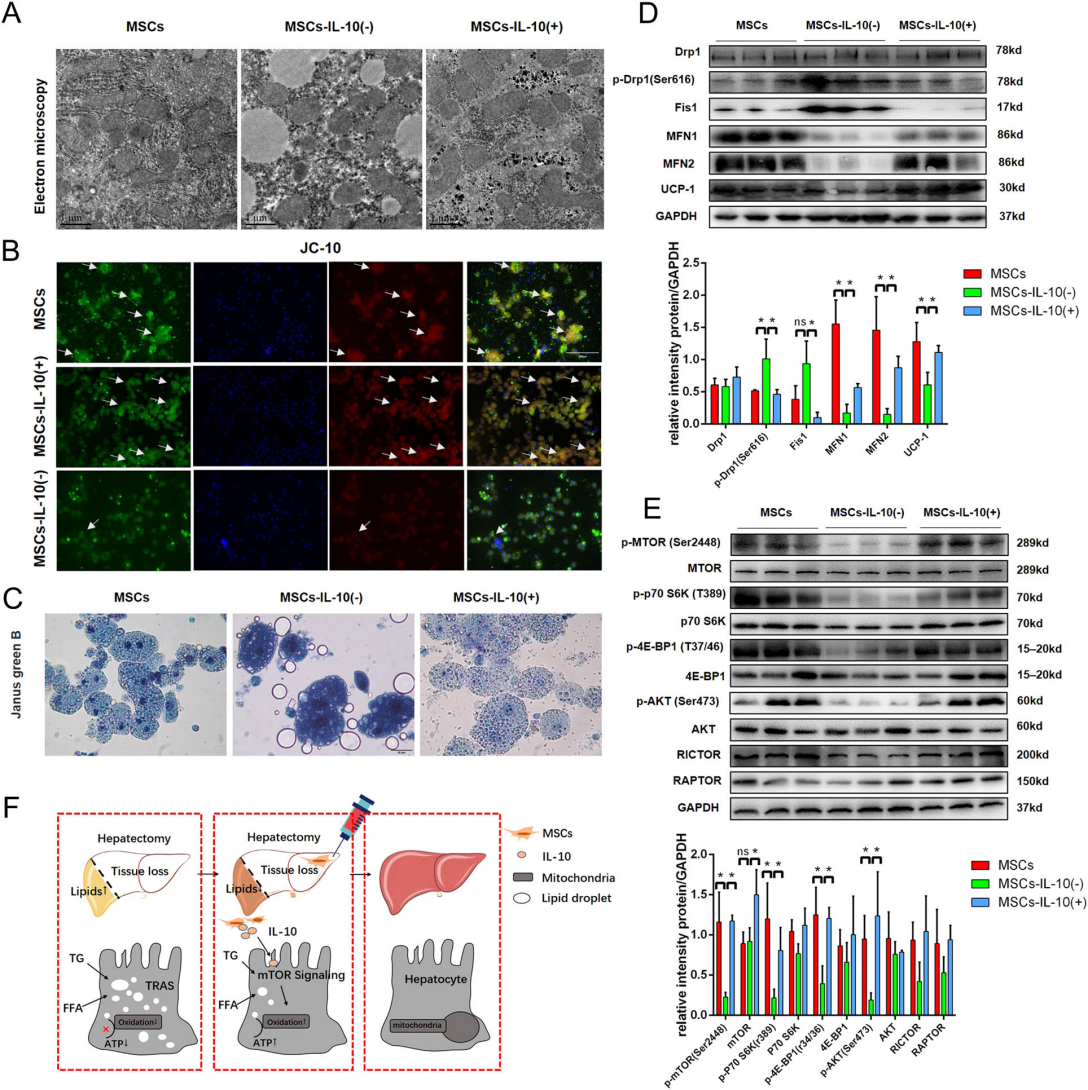
IL-10 is a key cytokine for MSCs to improve mitochondrial function in PHLF. **A** Observation of mitochondria in hepatocytes by transmission electron microscopy. **B** Observation of mitochondrial membrane potential in hepatocytes by JC-10 staining. **C** Janus green B staining. **D** Mitochondrial dynamic protein detection. **E** Western blotting analysis of mTOR pathway. **F** Graphical abstract. *P < 0.05. *t*-test, data are shown as mean standard deviation.

Considering the possible effect of IL-10 on lipid metabolism, we analyzed the lipid accumulation in the liver by oil red O staining and TEM. Oil red O staining suggested that there was more lipid accumulation in the MSCs-IL-10(−) transplantation group compared with the MSCs transplantation group, and more lipid droplets were observed in hepatocytes by TEM (Fig. 7A, B). As expected, the lipid accumulation in MSCs-IL-10(−) transplantation group was significantly higher compared with the other two groups (Fig. 7C). In order to explore the relationship between IL-10 and lipid accumulation in the liver, we examined the expressions of genes related to lipid metabolism. The results showed that IL-10 mainly affected the lipid catabolism-related genes (CPT1a, Hadha, and Hadhb) and lipid β oxidation-related proteins (PPARα, CPT1a, and CROT) (Fig. 7D, E). These results suggested that IL-10 was an important cytokine for MSCs to improve the mitochondrial damage in PHLF. Therefore, TEM was used to detect the morphology of mitochondria in the remaining liver of rats of three groups. The mitochondrial damage of the MCSs-IL-10(−) transplantation group was more serious compared with the other two groups (Fig. 8A). JC-10 staining and Janus green B staining also confirmed that IL-10 depletion reversed the repair effect of MSCs on mitochondrial damage in PHLF (Fig. 8B, C). Next, we tested the mitochondrial dynamic protein to further evaluate the effect of IL-10 on mitochondrial function. Our data showed found that the depletion of IL-10 counteracted the effect of MSCs on mitochondrial dynamics (Fig. 8D). The depletion of IL-10 could not only reverse the therapeutic effect of MSCs transplantation but also inhibit the activation of the mTOR pathway after MSCs transplantation (Fig. 8E). Therefore, MSCs could improve lipid metabolism mainly by secreting IL-10 and affecting mitochondrial function.

## Discussion

Dysfunction induced by loss of liver volume is a key factor in the steatosis of the remaining liver. Liver steatosis is characterized by the rapid accumulation of lipid droplets composed of TG and FA^25^. Zhang et al.^26^ have found that lipid accumulation in the liver activates macrophages through the p38 α pathway, promoting the release of TNF-α, CXCL10, and IL-6 to aggravate liver injury. Excessive lipid accumulation can induce liver lipotoxicity and directly activate UPR (unfolded protein response) to further damage hepatocytes^27^. However, the molecular correlation between PHLF and lipid metabolism still remains large unexplored. Our previous study has found that patients subjected to 90% hepatectomy have very serious steatosis. Moreover, lipid accumulation is accompanied by mitochondrial dysfunction. MSCs transplantation from the portal vein can effectively reduce the lipid accumulation and mitochondrial dysfunction after hepatectomy. The therapeutic effect of MSCs depends on paracrine cytokines, including IL-10, PGE2, and IL-6^28^. IL-10 is a cytokine that can regulate the growth and differentiation of innate immune cells^29^. At present, more and more studies have confirmed that IL-10 can regulate cell proliferation, apoptosis, angiogenesis, and inflammation^22,23^. Increasing evidence has shown that the therapeutic effect of MSCs depends on the secretion of IL-10^30^. However, the effect of IL-10 secretion by MSCs on PHLF remains unclear. Our study found that MSCs secreted IL-10 to alleviate liver failure after hepatectomy, and inhibition of IL-10 secretion could reverse the therapeutic effect of MSCs. Interestingly, a certain relationship seemed to exist between IL-10 and lipid metabolism or mitochondrial function. In the present study, we only briefly described such correlation, and more research is required to reveal the deep regulatory correlation between IL-10 and lipid metabolism.

Mitochondria have important roles in regulating lipid metabolism and oxidative stress in hepatocytes. The damage of mitochondrial function will lead to the inhibition of oxidative decomposition of free FA β in hepatocytes, increase the contents of lipid droplets in hepatocytes, and induce the fatty degeneration of hepatocytes^31^. In the case of energy deficiency or mitochondrial damage, mitochondria maintain their quantity and quality through the biosynthesis process^32^. After hepatectomy, with the aggravation of lipid accumulation in hepatocytes, the mitochondrial β-oxidation capacity and mitochondrial uncoupling are gradually decreased^33^. Subsequently, oxidative stress, lipid peroxidation, and oxidative DNA damage are associated with decreased antioxidant capacity and increased inflammation, leading to mitochondrial dysfunction^34^. Meanwhile, under the effects of lipid peroxidation products and TNF-α on mitochondrial respiration, mitochondrial ROS oxidizes unsaturated FAs to cause lipid peroxidation. Lipid accumulation after hepatectomy can damage the ultrastructure of mitochondria and change the mitochondrial dynamics, thus reducing the activity of mitochondrial respiratory chain complex and β-oxidation function^35^. The damage of mitochondrial function further aggravates the steatosis after hepatectomy by reducing the β-oxidation of FA, forming a vicious circle, which is difficult to correct. Moreover, the decreased synthesis of damaged mitochondria and ATP affects the energy supply needed for liver regeneration and aggravates the PHLF^36^.

mTOR is a major growth and metabolism regulatory pathway, which can sense different nutritional statuses and environmental factors, including growth factor, energy level, and cell metabolism^37^. Recent results have shown that mTORc1-s6k can promote lipid oxidation and reduce lipid accumulation in the liver^38^. We examined the mTOR pathway and found that the mTOR pathway was affected by MSCs transplantation. MSCs activated the mTOR pathway by secreting IL-10. Activation of the mTOR pathway is critical for liver regeneration after hepatectomy^39^. Park’s investigation found that inhibiting the mTOR pathway by using RAP can cause apoptosis, but this process can be reversed by supplementing IL-10^40^. In our experiment, the mTOR pathway in hepatocytes was activated by enhancing the secretion of IL-10 by MSCs. IL-10, an immunomodulatory factor, seems to have a positive regulatory effect on the mTOR pathway in the post-hepatectomy state. IL-10 can regulate lipid metabolism by affecting mitochondrial respiratory function. As an immunomodulatory factor, the function of regulating metabolic balance is easy to be ignored^41^. Through electron microscopy and oil red staining, we found that IL-10 secreted by MSCs can affect liver steatosis induced by hepatectomy. IL-10 secreted by MSC corrected the imbalance of mitochondrial dynamics and promoted the maintenance of mitochondrial function. Protect the remaining liver from lipid toxicity caused by excessive lipid accumulation. It was worth considering that the mTOR pathway was closely related to autophagy, and the therapeutic effect of MSCs was related to mitochondrial function. Interestingly, the level of autophagy was reduced in the MSCs group compared with the control group. It seems that MSCs does not have a therapeutic role through lipophagy (Supplementary Fig. 1C). We will further explore the relationship between mitochondrial autophagy and MSCs in the PHLF model.

Collectively, we evaluated the efficacy of MSCs in the treatment of liver failure induced by 90% hepatectomy. The therapeutic effect of MSCs transplantation from the portal vein was confirmed. Moreover, we briefly discussed the relationship between MSCs and lipid accumulation or mitochondrial function in hepatectomy. The role of IL-10 in these factors was clarified. Finally, we concluded that MSCs might be an effective therapeutic regimen for PHLF.

## Supplementary information

Supplementary Figure 1

## Data Availability

The data sets generated during and/or analyzed during the current study are available from the corresponding author on reasonable request.
