# Supplementary figures and images for: Mesenchymal stem cells ameliorate lipid metabolism through reducing mitochondrial damage of hepatocytes in the treatment of post-hepatectomy liver failure
Source: Cell Death Dis. 2021 Jan 21;12(1):111. doi: 10.1038/s41419-020-03374-0 (PMC7820227; doi:10.1038/s41419-020-03374-0)

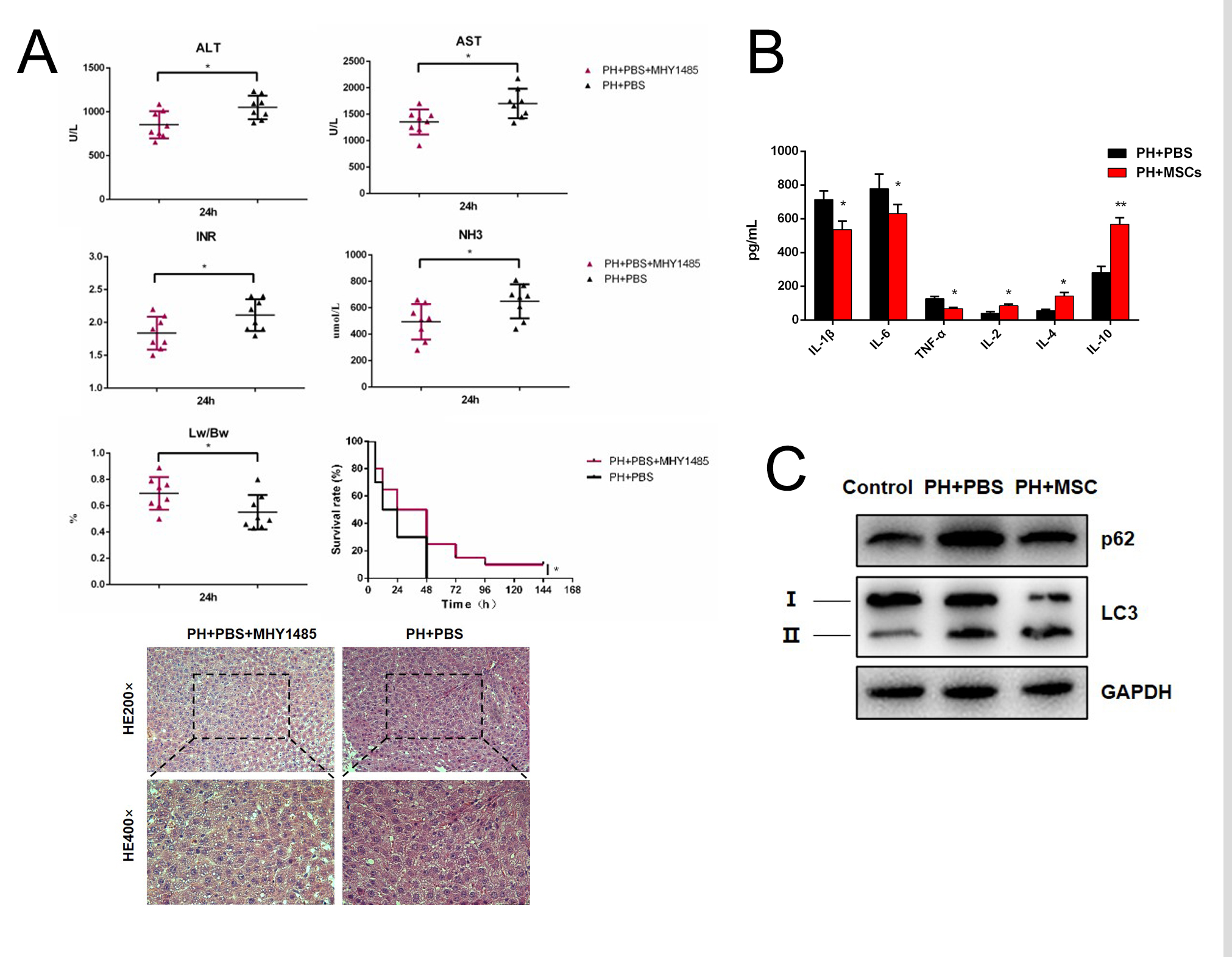

Supplement: Supplementary file 1 — Supplementary Figure 1 [file 41419_2020_3374_MOESM1_ESM.jpg]
